# A Case of Orbital Apex Syndrome Related to Herpes Zoster Ophtalmicus

**DOI:** 10.7759/cureus.27254

**Published:** 2022-07-25

**Authors:** Atsuki Fukushima, Masaki Mihoshi, Yukiko Shimizu, Hitoshi Tabuchi

**Affiliations:** 1 Ophthalmology, Tsukazaki Hospital, Himeji, JPN; 2 Ophthalmology, Hiroshima University, Hiroshima, JPN

**Keywords:** ptosis, varicella zoster virus, eye movement, orbital apex syndrome, herpes zoster ophthalmicus

## Abstract

Orbital apex syndrome (OAS) is a rare disease. One of the causes of OAS is herpes zoster ophthalmicus (HZO). A 73-year-old man developed herpes zoster around the right eye, and oral amenamevir treatment was given for seven days. The right eyelid ptosis was observed on the third day, and right eye movement was restricted in all directions on the ninth day. His eyesight was also poor, and he was diagnosed with OAS associated with HZO. Cerebrospinal fluid examination revealed mononuclear cell increase; however, VZV-DNA was not detected. Intravenous infusion of acyclovir and oral prednisolone administration were started. Two weeks after the start of treatment, ptosis, eye movements, and visual acuity improved. If HZO is found, it is necessary to consider the possibility of developing OAS.

## Introduction

Herpes zoster results from reactivation of the varicella zoster virus (VZV) and has been increasing in recent years [1]. Reactivation occurs more than decades after the initial infection. Suppressed immune responses due to such as aging and diabetes are considered to be the reason for reactivation of VZV [1]. Although it is found in various parts of the body with painful cutaneous vesicules, if it is found on the face, especially around the eyes, it is treated as herpes zoster ophthalmicus (HZO). Regarding HZO, ophthalmologists usually pay attention to blepharitis, conjunctivitis, keratitis, iritis, and retinal necrosis. Orbital apex syndrome (OAS), which leads to ptosis, ocular motility disorder, and optic nerve system disorder, is caused by tumor, vascular diseases, inflammation, and infection [2]. Although few reports are available, VZV may spread to the orbital part, damage various cranial nerves, and cause OAS [3-12]. Regarding ocular motility disorder, VZV spreads through the traffic branches between the first branch of the trigeminal nerve, and an oculomotor nerve, a trochlear nerve, and an abducens nerve [13].

## Case presentation

**Figure 1:**
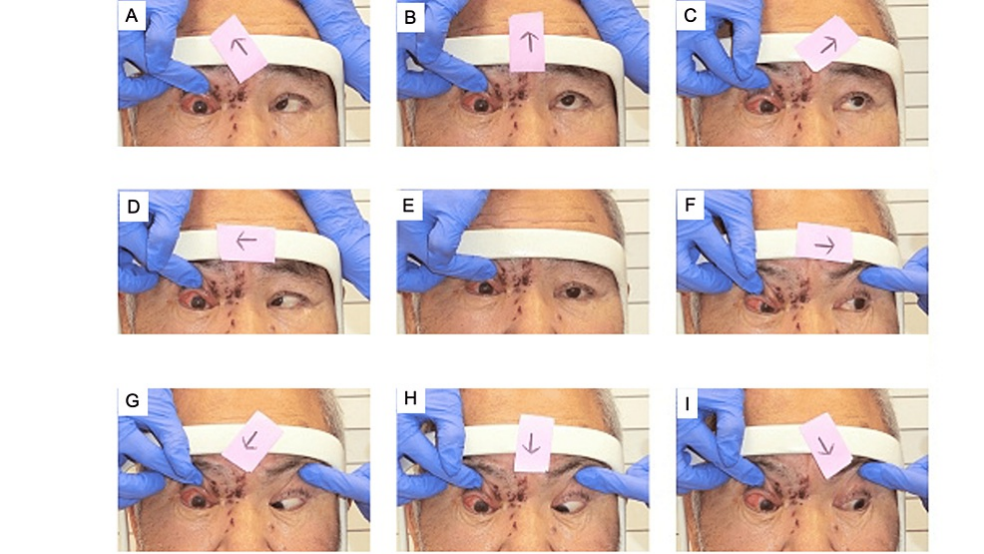
Nine-way eye position on June 3, 2022. (A) Right upper view. (B) Upper view. (C) Left upper view. (D) Right view. (E) Front view. (F) Left view. (G) Right lower view. (H) Lower view. (I) Left lower view. Eye movement was completely inhibited.

The case is of a 73-year-old male. On May 25, 2022, he noticed swelling of the upper right eyelid. He visited a nearby clinic and was diagnosed with HZO. From May 27, he was prescribed amenamevir 400 mg for one week and acyclovir eye ointment three times a day. From May 28, he became aware of ptosis and was introduced to the Department of Ophthalmology at Tsukazaki Hospital on June 3. Right eyelid ptosis and right omnidirectional eye movement restriction were noted (Figure 1).

**Figure 2:**
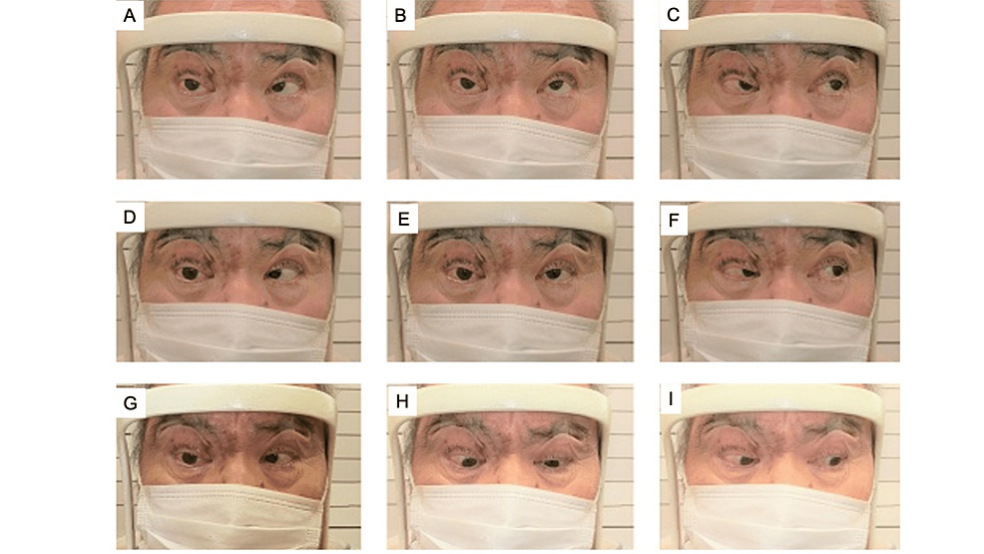
Nine-way eye position on June 20, 2022. (A) Right upper view. (B) Upper view. (C) Left upper view. (D) Right view. (E) Front view. (F) Left view. (G) Right lower view. (H) Lower view. (I) Left lower view. Eye movement improved compared to Figure 1.

In addition to Hutchinson's sign, conjunctival hyperemia, corneal epithelial disorder, and iritis were observed in the right eye, but retinal necrosis was not observed. The right corrected visual acuity was 0.1, and the critical fusion frequency was 34 Hz on the right eye and 41 Hz on the left eye. On the same day, he was hospitalized and underwent a cerebrospinal fluid test, which confirmed mononuclear cell predominant pleocytosis (120 cells/mL, mononuclear cells: 96.7%) and an increase in varicella-shaped herpes IgG antibody in the cerebrospinal fluid. Cerebrospinal fluid PCR did not detect VZV DNA. No abnormal findings were found on head and orbital MRI examinations. After admission, intravenous infusion of acyclovir 750 mg was started and terminated for eight consecutive days. In addition, prednisolone 30 mg was orally administered for nine days. Then, the dose of prednisolone was reduced to 25 mg. One week later, ptosis, ocular motility disorder (Figure 2), and the right corrected visual acuity (0.2) improved, and treatment is planned to be continued.

## Discussion

The number of English reports regarding OAS by HZO is not abundant [3-12]. The reason may be that antiviral treatment against VZV prevents the development of OAS in many cases.

In our case, anti-VZV antibody was confirmed in the cerebrospinal fluid, but VZV DNA was not detected. This may be due to the fact that 10 days have passed since the onset and that he had been receiving amenamevir treatment for one week. Furthermore, abnormal findings were not found on MRI. However, ocular motility disorder was noted. It could be possible that VZV have spread along the traffic branches of the interneuron, as reported before [13].

HZO is initiated by VZV infection, and then cellular immunity against VZV follows and induces immune-mediated pathologies in the central nervous system [6-10]. Thus, suppression of immune responses by steroids may be necessary if excessive immune responses occur. Although there are pros and cons regarding the administration of steroids for HZO, antiviral treatment and steroid treatment were reported to be effective [4,7-9]. In our case, antiviral treatment had been conducted for seven days before hospitalization and the blisters disappeared and turned into scabs, indicating that the skin symptoms had subsided. Therefore, we decided to use systemic steroid in addition to antiviral treatment. These regimens appeared to be effective, and the course of this case is thought to gradually improve similar to previous reports [4,7-9].

If VZV DNA is not detected in the cerebrospinal fluid such as in our case, concomitant use of steroids may be more effective to suppress excessive immune responses against VZV.

## Conclusions

It is necessary to consider the possibility of OAS development in HZO. Furthermore, properly grasping the status of viral inflammation by cerebrospinal fluid examination and MRI examination will help select a treatment method.
